# Impact of Subcutaneous Versus Orthotopic Implantations on Patient-Derived Xenograft Transcriptomic Profiles

**DOI:** 10.1158/2767-9764.CRC-25-0008

**Published:** 2025-05-28

**Authors:** Yanghui Sheng, Zixuan Xie, Jingjing Wang, Xueying Yang, Mengtian Yao, Wubin Qian, Likun Zhang, Xiaobo Chen, Sheng Guo

**Affiliations:** Crown Bioscience Inc., Suzhou, China.

## Abstract

Patient-derived xenografts (PDX) are essential preclinical models, capturing the histologic and molecular features of human tumors. Subcutaneous (s.c.) and orthotopic (ortho) PDXs are widely used, but their comparative utility remains unclear, especially regarding tumor and stromal gene expression. This study analyzed 45 matched s.c. and ortho PDX models spanning five cancer types using bulk RNA sequencing. Tumor (human) gene expression was highly conserved between s.c. and ortho PDXs, with similar epithelial–mesenchymal transition, angiogenesis, and stemness scores. In contrast, stromal (mouse) gene expression varied by implantation site, with ortho models better reflecting native tissue environments. A conserved subset of stromal genes, consisting of histone and ribosomal protein genes and driven by tumor-intrinsic factors, exhibited stable expression patterns across implantation sites, indicating that tumor characteristics shape stromal responses. Differential expression analysis identified metastasis-related stromal genes in both PDX types, although no direct links to metastatic pathways were found. These findings highlight the stability of tumor-driven gene expression across implantation sites and reveal the impact of implantation location on stromal profiles, guiding model selection for future cancer research.

**Significance:**

This study reveals conserved tumor gene expression, distinct tumor microenvironment differences, and key stromal and metastasis-related genes in s.c. and ortho PDX models, providing valuable insights for oncology drug development.

## Introduction

The translational impact of a preclinical study in oncology hinges on selecting a disease model that faithfully represents the complexity of human cancer. Patient-derived xenografts (PDX), which closely recapitulate the histologic features, molecular characteristics, and drug responsiveness of patient tumors (1–3), are currently regarded as the leading preclinical model system in oncology. Notably, the incorporation of large, well-characterized PDX collections—complete with genomic and transcriptomic data—has greatly facilitated the discovery and validation of novel biomarkers for treatment response (4–7). These resources play an increasingly important role in guiding personalized medicine.

PDX models are typically generated by engrafting patient tumor specimens into immunocompromised mice using subcutaneous (s.c.), orthotopic (ortho), or heterotopic implantation techniques (5). S.c. implantation is the most commonly used because it is technically straightforward and enables easy longitudinal tumor volume measurements using calipers, thereby permitting the applications of various statistical tools to quantitatively evaluate treatment efficacy (8–12). However, whereas s.c. PDXs offer practical advantages, the majority of these tumors fail to metastasize (13), limiting their utility in studying metastatic processes. Ortho PDX models, by contrast, better mimic the native tumor environment (TME) and can recapitulate the metastatic process from the correct anatomic site (13). Although the development of ortho PDXs dates back to the 1990s, and established protocols exist for major tumor types, creating ortho models and monitoring tumor growth in deep tissues often remain technically challenging. These procedures typically require complex microsurgical techniques and advanced imaging technologies, which limit their scalability and throughput (14–21). Thus, there is no absolute consensus as to which approach is superior. The choice between s.c. and ortho PDXs depends on the specific research question, with each model type offering unique strengths.

In addition to these established considerations, another critical factor influencing model selection is the expression level of specific target genes. Although traditional cancer therapies focus on eradicating malignant cancer cells, the crucial role of the TME in shaping disease progression and treatment response has gained increasing recognition (22). This understanding has spurred interest in interventions aimed at the tumor stroma. Despite this focus, key questions remain: for a given cancer type, how do the stromal cell populations and their gene expression patterns differ between s.c. and ortho TMEs? Furthermore, does the site of implantation significantly influence gene expression profiles in the malignant cancer cell component?

To address these knowledge gaps, we performed bulk RNA-sequencing (RNA-seq) on 45 PDX models spanning five cancer types, each represented by matched ortho/s.c. tumor samples. Our analyses revealed that the overall gene expression profiles of cancer cells were highly similar between the s.c. and ortho counterparts of the same PDX models. Moreover, no metastasis-related enriched Gene Ontology (GO) terms were preferentially enriched in either the mouse or human components of the ortho PDXs compared with their matched s.c. PDXs. Taken together, these findings provide clearer guidance on selecting the appropriate PDX models for future preclinical research, enabling investigators to more strategically align experimental design with translational objectives.

## Materials and Methods

### PDX model establishment

The 45 PDX models, including breast, colorectal, gastric, pancreatic, and liver cancers, were developed and established with immune-deficient mice in Crown Bioscience under the approval of the institutional review boards of the hospitals and informed consent from patients. Written informed consent was obtained from all patients, and the study was conducted in accordance with the Declaration of Helsinki and approved by the Institutional Review Board. Cryopreserved or fresh tumor tissues were cut into small pieces (approximately 2–3 mm in diameter) and then subcutaneously transplanted in the right flank of NOD/SCID or BALB/c nude mice, or orthotopically engrafted into the mice mammary fat pad (for breast cancer PDXs) or hepatic parenchyma of the left lobe of the liver (for liver cancer PDXs) or immobilized into the wall of the stomach (for gastric cancer PDXs) or pancreas (for pancreatic cancer PDXs) or the wall of the cecum (for colorectal cancer PDXs) by absorbable surgical sutures, for tumor development. Primary tumors were used in subsequent analyses for both s.c. and ortho implantations. All the animal study procedures were performed in the specific pathogen-free animal facility at Crown Bioscience under the approved protocols by the Institutional Animal Care and Use Committee, with the guidance of the Association for Assessment and Accreditation of Laboratory Animal Care.

### Bulk RNA-seq

RNA was extracted from the PDX models using Qiagen RNeasy Mini Kit. The purified RNA was then quantified using a NanoDrop 2000 spectrophotometer (Thermo Fisher Scientific) and further assessed for quality with an Agilent 4200 TapeStation. Subsequently, RNA libraries were constructed using the TruSeq RNA Sample Prep Set (Illumina). Poly-A mRNA was first purified from total RNA using oligo-dT–attached magnetic beads and fragmented. Subsequently, the first-strand and second-strand cDNA were synthesized. Following cDNA cleanup, end-repair, A-tailing, adapter ligation, PCR amplification, and purification were carried out according to the library construction protocol. The concentration of the resulting RNA library was measured using a Qubit 3.0 fluorometer with the dsDNA HS Assay (Thermo Fisher Scientific), whereas the size distribution was analyzed using an Agilent 4200 TapeStation. Finally, bulk RNA-seq was performed on the NovaSeq 6000 platform.

### RNA-seq data processing

The quality of raw RNA-seq data was assessed using FastQC v0.11.3 (RRID: SCR_014583; ref. 23). Sequencing adapters and low-quality sequences were removed with Trimmomatic v0.36 (RRID: SCR_011848; ref. 24). Cleaned reads were then mapped to human (hg19) and mouse (mm10) reference genomes using STAR v2.5.1b (25), enabling separation of human and mouse components. Reads mapping to both reference genomes were assigned based on the alignment with fewer mismatches, with ties classified as human reads. Gene expression levels were quantified using pseudoalignment in Kallisto 0.42.5 (26). Transcriptome indices were generated from the University of California, Santa Cruz GENCODE v24 asic gene set for human and the mm10 annotation for mouse. This workflow provided accurate and computationally efficient transcript quantification while distinguishing between species in mixed-species RNA-seq data.

### Calculation of gene set enrichment scores

Enrichment scores for epithelial–mesenchymal transition (EMT), angiogenesis, stemness, and stromal and immune gene signatures were calculated for the human and mouse components of each sample based on transcript abundance data in transcripts per million. EMT scores were computed using the BioQC package (27), leveraging the wmwTest() function, which supports signed gene sets. This method utilizes two gene sets: one representing positively regulated mesenchymal signatures and the other negatively regulated epithelial signatures, as defined in a previously published pan-cancer study (28). Stemness and angiogenesis scores were calculated using the gene set variation analysis method, implemented in an R package (29). The stemness gene set was derived from a previous study (30), and the angiogenesis gene set was obtained from MSigDB (31). Stromal and immune scores were calculated using the R package *ESTIMATE* (32).

### Differential expression analysis

Transcript-level read counts obtained from Kallisto were used as inputs for differential expression analysis using the *sleuth* package in R (33). Separate analyses were conducted for the human and mouse components, using ortho PDXs as the reference for both. Gene-level differential expression analysis was performed by aggregating the transcript-level counts into gene-level counts using the sleuth_prep() function with the gene_mode parameter set to TRUE. The analyses used the default filters of *sleuth* and utilized the Wald test.

### Data availability

The raw RNA-seq data in FASTQ format have been deposited in the NCBI Sequence Read Archive under the accession number PRJNA1205404 (https://www.ncbi.nlm.nih.gov/sra/PRJNA1205404).

## Results

### Tissue-specific stromal and cancer gene expression dynamics in ortho versus s.c. PDX models

We performed bulk RNA-seq on 90 PDX tumor samples, comprising 45 pairs of ortho and s.c. implants (Supplementary Table S1). Sequencing reads were sorted into human and mouse components, corresponding to the cancer cell and stroma cell transcriptomes, respectively. In a subset of PDX samples, the mouse component yielded low read counts and uneven read coverage, as indicated by low median transcript integrity scores (Supplementary Fig. S1A; ref. 34). Because accurate transcript quantification relies on even read coverage, we excluded all samples with median transcript integrity scores below 65 from subsequent analysis.

**Figure 1:**
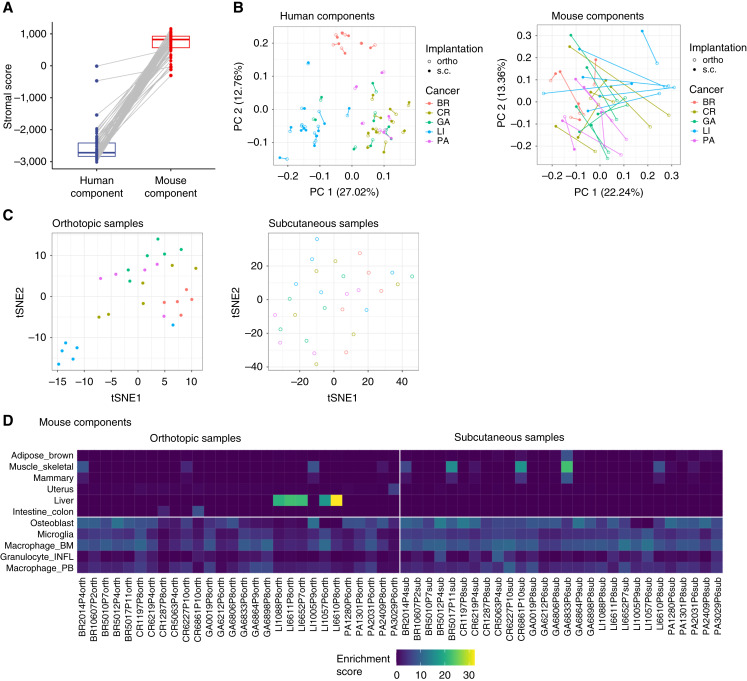
Overview of the characteristics of ortho and s.c. PDX models. **A,** Comparison of ESTIMATE stromal scores between the human (cancer) and mouse (stromal) transcriptomes. **B,** Principal component (PC) analysis of gene expression profiles for both human and mouse components. Data points are shaped according to the implantation method (ortho vs. s.c.) and colored by cancer type. **C,***t*-distributed stochastic neighbor embedding (*t*-SNE) visualization of mouse (stromal) components from both ortho and s.c. PDX samples, colored by cancer type. **D,** Tissue-dependent clustering of the mouse (stromal) components from ortho and s.c. PDX samples, with color gradients representing enrichment scores. BM, bone marrow; BR, breast; CR, colorectal; GA, gastric; INFL, inflammatory; PA, pancreatic; PB, peripheral blood.

For most of the remaining samples, the ESTIMATE stromal scores (32), which reflect similarity to established stromal expression profiles, were much higher in the mouse component than in the human component (Fig. 1A). This confirmed the high species-specificity of our read sorting procedure. However, two outlier samples, both from the same PDX model (LI3205P5), displayed comparable stromal scores in the human and mouse fractions. This finding could reflect either retention of patient-derived stromal cells in that model (6) or unusually high expression of stromal marker genes within the cancer cells themselves.

Principal component analysis demonstrated that gene expression profiles of the human (tumor) components clustered primarily by cancer type, with individual ortho/s.c. pairs from the same PDX model grouping closely together (Fig. 1B). In contrast, the mouse (stromal) components did not show clear clustering by cancer type, and the spatial proximity of ortho/s.c. sample pairs varied considerably (Fig. 1B). Interestingly, when we applied *t*-distributed stochastic neighbor embedding to the mouse (stromal) components, the ortho samples formed cancer type–dependent clusters, whereas the s.c. samples did not (Fig. 1C). This pattern suggests that the transcriptomic profiles of the stromal components in PDX models are influenced by the implantation site.

Further analysis using the BioQC package (27) identified a strong enrichment of liver-specific genes in five of six ortho liver (LI) samples (Fig. 1D, top left; adjusted Wilcoxon–Mann–Whitney *P* values < 1e−16), potentially reflecting the inclusion of adjacent liver tissue during sample dissection. Additionally, we detected a global enrichment of immune cell signatures in both ortho and s.c. samples (Fig. 1D, bottom), consistent with the presence of immune cells in the TME.

### Distinct correlation patterns between tumor and stromal gene expression across PDX implantation sites

**Figure 2:**
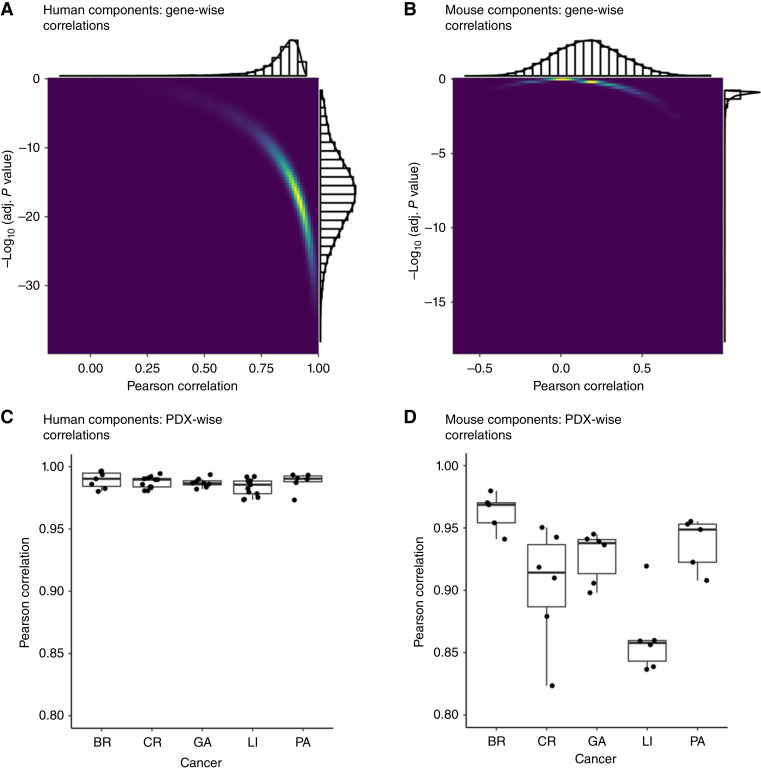
Gene-level and PDX-level correlation patterns. **A,** For the human (cancer) component, a color-density plot and accompanying histograms showing log_10_ adjusted *P* values plotted against gene-wise ortho-vs-s.c. Pearson correlation coefficients. **B,** For the mouse (stromal) component, a color-density plot and accompanying histograms displaying log_10_ adjusted *P* values vs. gene-wise ortho-vs-s.c. Pearson correlation coefficients. **C,** A boxplot of PDX-wise ortho-vs-s.c. correlation coefficients by cancer type for the human component, showing consistently high correlations across cancer types. **D,** A boxplot of PDX-wise ortho-vs-s.c. correlation coefficients by cancer type for the mouse component. BR, breast; CR, colorectal; GA, gastric; PA, pancreatic.

To characterize pairwise correlation patterns between the expression profiles of ortho and s.c. PDX samples, we split the human- and mouse-component expression matrices into two submatrices, one for s.c. samples and one for ortho samples, and computed the pairwise Pearson correlation coefficients at both the gene and PDX levels (Supplementary Fig. S1B). In the human component, gene-wise ortho-vs-s.c. Pearson correlation coefficients are predominantly greater than 0.5, with highly significant adjusted *P* values (Fig. 2A). In contrast, the distribution of gene-wise ortho-vs-s.c. Pearson correlation coefficients in the mouse component were much broader (Fig. 2B), and only a small proportion of mouse genes (711 of 14,217) exhibited significantly positive correlations (adjusted *P* values < 0.05). At the PDX level, ortho-vs-s.c. Pearson correlations in the human component were uniformly high (above 0.97), indicating that the overall gene expression patterns are highly similar between the ortho sample and the s.c. sample across all PDX models examined (Fig. 2C). By comparison, the PDX-level correlations in the mouse component are much more variable, especially among the colorectal and LI models (Fig. 2D).

### A conserved subset of stromal genes driven by tumor-intrinsic factors

As noted above, we identified a small subset of mouse genes (∼2%) whose expression levels are significantly correlated between the stromal components of s.c. and ortho PDX models (Supplementary Table S2). Rather than implying identical absolute expression levels of these mouse stromal genes across all PDX lines, this positive correlation indicates that PDX models with comparatively higher expression of these genes in the stromal component under one implantation condition tend to exhibit similarly higher expression under the other. In other words, whereas the absolute expression levels can vary, the differences between individual PDX models remain consistent, suggesting that tumor-intrinsic factors shape a stable stromal gene expression profile across distinct microenvironments.

This subset includes histone genes (e.g., *H2ac15*, *H2ac20*, and *H2ac13*) and ribosomal protein genes (e.g., *Rps2* and *Rps12*–*13*), which are typically considered “housekeeping” due to their central roles in cellular function. In this context, however, their robust inter-model correlations across implantation sites point to a core set of tumor-driven stromal responses that persist even when the tumor grows in different tissues.

To gain further insight into the roles of these genes, we performed GO enrichment analyses, identifying 47 enriched biological processes (Supplementary Table S3). Most were related to morphogenesis, tissue patterning, and developmental signaling, reinforcing the notion that tumor–stroma interactions rely on a conserved set of molecular mechanisms. These findings underscore how intrinsic tumor properties can establish and maintain a stable stromal gene expression program, supporting tumor growth regardless of implantation site.

### Tumor-intrinsic stability versus stromal variability in EMT, angiogenesis, and stemness

EMT, angiogenesis, and stemness are critical processes driving tumor progression, invasion, metastasis, and resistance to conventional therapies (35–38). These processes are not solely intrinsic to cancer cells but are also profoundly influenced by the TME, which includes a diverse array of stromal and immune cells. Because the composition and activity of the TME shape the extent of EMT, angiogenesis, and stemness, assessing the abundance of stromal and immune components provides valuable insight into these processes. To estimate the contribution of these components, we computed ESTIMATE stromal and immune scores separately for the human (tumor) and mouse (stromal) compartments and assessed correlations between s.c. and ortho PDX samples using Pearson correlation.

**Figure 3:**
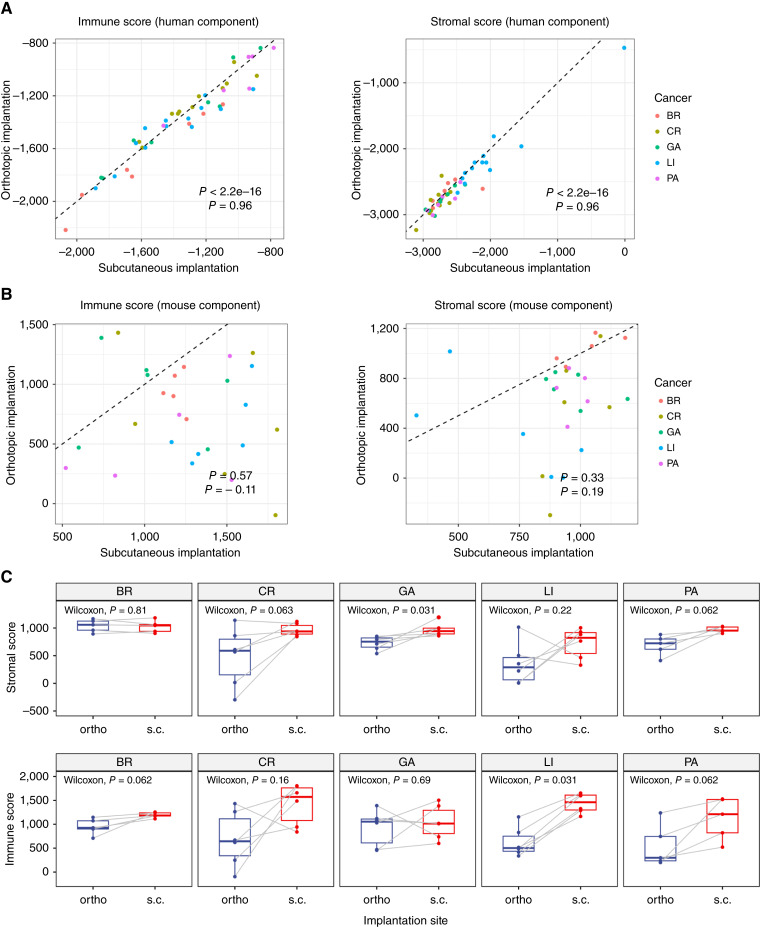
ESTIMATE immune and stromal scores in ortho vs. s.c. PDX models. **A,** Pearson correlation analysis of ESTIMATE immune scores and stromal scores in the human components between ortho and s.c. PDX models, with colors representing different cancer types. The dashed line indicates the line of equality (*y* = *x*). **B,** Pearson correlation analysis of ESTIMATE immune scores and stromal scores in the mouse components between ortho and s.c. PDX models, with colors representing different cancer types. The dashed line indicates the line of equality (*y* = *x*). **C,** Paired Wilcoxon tests comparing ESTIMATE immune scores and stromal scores in the mouse component between ortho and s.c. PDX models, stratified by cancer types, with colors representing the implantation methods. BR, breast; CR, colorectal; GA, gastric; PA, pancreatic.

In the human (tumor) component, both immune and stromal scores showed strong correlations between s.c. and ortho pairs (Fig. 3A). In contrast, the mouse (stromal) component showed no such correlation (Fig. 3B), suggesting that stromal and immune cell abundance depends heavily on the implantation site. To investigate whether this inconsistency is cancer type–dependent, we further compared the stromal and immune scores between the two PDX types within each cancer type using the paired Wilcoxon test. The results showed varying levels of inconsistency across all five cancer types, reinforcing the site-specific nature of the mouse stromal expression patterns (Fig. 3C).

An MCP-counter analysis was further performed to compare specific cell types of tumor and stromal components in different implantation sites. In the human (tumor) component, MCP-counter analysis revealed strong correlations in various immune and stromal cell scores between s.c. and ortho pairs, consistent with the previously observed ESTIMATE results (Supplementary Fig. S2A). Notably, T cell, B lineage, NK cell, myeloid, and fibroblast scores all showed significant correlations, suggesting that the immune and stromal landscape within the tumor compartment is relatively conserved across implantation sites. In contrast, the mouse (stromal) component displayed weaker or no correlations in corresponding cell scores (Supplementary Fig. S2B). Whereas certain cell types, such as fibroblasts, showed moderate correlation, others like T cells and B lineage cells exhibited minimal correlation. These findings again support the notion that stromal and immune cell abundance within the mouse component is influenced by the implantation site (39).

**Figure 4:**
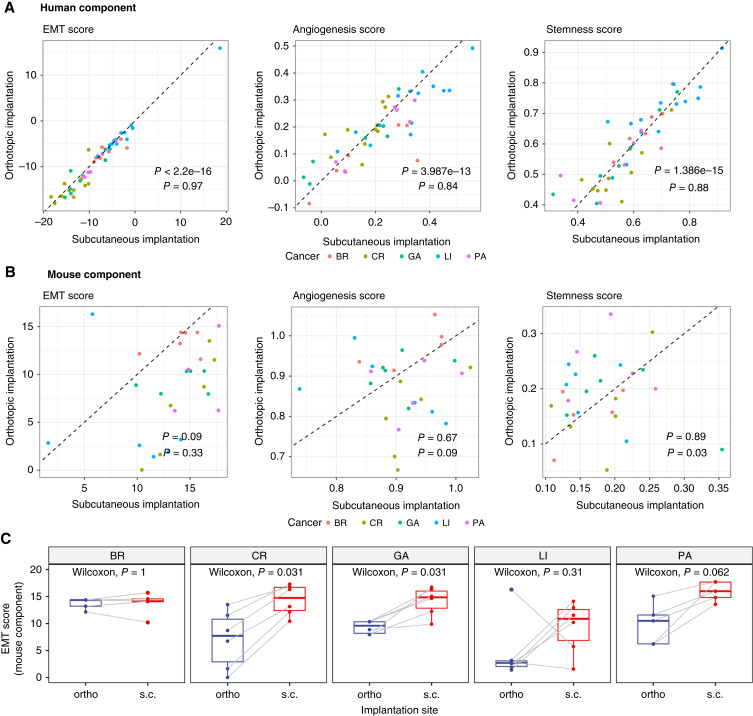
EMT, angiogenesis, and stemness scores in ortho vs. s.c. PDX models. **A,** Pearson correlation analysis of EMT, angiogenesis, and stemness scores in the human component between ortho and s.c. PDX models. Colors represent different cancer types. The dashed line indicates the line of equality (*y* = *x*). **B,** Pearson correlation analysis of EMT, angiogenesis, and stemness scores in the mouse component between ortho and s.c. PDX models, with colors representing different cancer types. The dashed line indicates the line of equality (*y* = *x*). **C,** Paired Wilcoxon tests comparing EMT scores in the mouse component between ortho and s.c. PDX models, stratified by cancer types, with colors representing the implantation methods. BR, breast; CR, colorectal; GA, gastric; PA, pancreatic.

We next calculated EMT, angiogenesis, and stemness scores for the human and mouse components of all PDX models and compared s.c./ortho pairs using Pearson correlation. In the human (tumor) component, all three scores showed strong, significant correlations (Fig. 4A), indicating that these tumor-associated processes remain stable across implantation sites. However, these scores were much less consistent in the mouse (stromal) component (Fig. 4B), reflecting a context-dependent stromal expression pattern influenced by the implantation site.

To explore whether the variability in stromal EMT scores is cancer type–dependent, we performed paired Wilcoxon tests comparing s.c. and ortho samples within each cancer type. EMT scores did not significantly differ by implantation type in BR models, whereas colorectal, gastric, LI (with one outlier), and pancreatic models displayed varying levels of inconsistency (Fig. 4C).

Overall, these findings highlight a striking similarity in tumor-associated processes within the human (tumor) component across implantation sites, whereas the mouse (stromal) component exhibits greater implantation site–specific variability. This underscores the stability of intrinsic tumor-driven programs, even when tumors are grown in distinct TMEs.

### Differential gene expression analysis identifies stromal genes potentially related to metastasis

**Figure 5:**
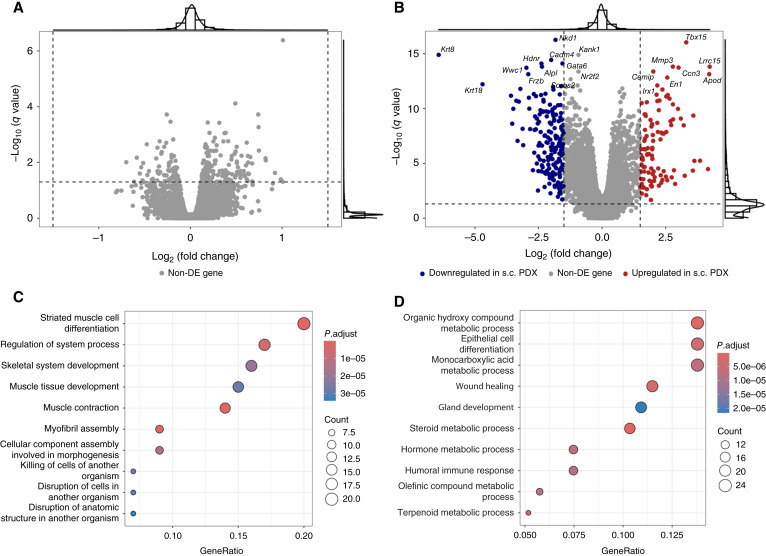
Differential expression between ortho and s.c. PDX models. **A,** Volcano plot displaying the results of DEG analysis in the human component. No DEGs were identified. **B,** Volcano plot displaying the DEGs identified in the mouse components, with colors indicating the direction of regulation. The horizontal and vertical dashed lines represent the *q*-value and log_2_ fold change thresholds, respectively. Non-DE, not differentially expressed. **C,** The top 10 significantly enriched GO terms among the upregulated DEGs in the mouse component of s.c. PDX models. **D,** The top 10 significantly enriched GO terms among the upregulated DEGs in the mouse component of ortho PDX models.

To further explore expression differences between ortho and s.c. PDX models, we performed differential expression gene (DEG) analysis on the human component of each s.c./ortho pair. Using a cutoff of *q*-value < 0.05 and log_2_ fold change > 1.5, we identified no DEGs (Fig. 5A), suggesting that the tumor cells from the two PDX types exhibit highly similar transcriptomic expression profiles.

To assess whether the surrounding stromal tissue might contribute to the observed differences in metastatic behavior between the two PDX model types, we conducted the same DEG analysis on the mouse component. Applying the same cutoff (*q*-value < 0.05 and log_2_ fold change > 1.5), we identified 287 DEGs (Fig. 5B; Supplementary Table S4). Among these, we found several metastasis-associated genes upregulated in one PDX type. For instance, *Ceacam1*, *Wnt4*, and *Erbb3*, which promote metastasis by enhancing cellular motility and reducing adhesion, showed higher expression in ortho PDX models compared with their s.c. counterparts (40–42). Conversely, genes like *Mmp3*, *Six1*, and *Aspn*, previously linked to metastatic processes in various cancers, were upregulated in s.c. PDX models (43–45). This finding suggests that s.c. implantation may still retain certain metastasis-associated stromal characteristics at the gene expression level.

To gain deeper insight and evaluate whether these DEG differences are linked to metastasis-related functions, we performed GO enrichment analysis on the upregulated DEGs identified in both the mouse components for both PDX models. However, none of the significantly enriched GO terms were directly related to metastatic processes (Fig. 5C and D). The complete results of the GO enrichment analysis are provided in Supplementary Tables S5 and S6.

## Discussion

Since their introduction, the specific applications of ortho versus s.c. PDX models have remained somewhat unclear. In this study, we comprehensively analyzed differences in gene expression profiles between these two implantation types. To minimize the confounding effects of murine stromal contamination in PDX tumor tissues (46), we carefully separated human and mouse reads during post-alignment processing. This step, taken before conducting differential gene expression analyses, enabled a more precise comparison between the two PDX model types.

Our findings revealed strong correlations in gene expression profiles, EMT scores, and both stromal and immune scores within the human (tumor) component of the ortho and s.c. PDX models. These results suggest that, based on transcriptomic analysis, the intrinsic gene expression programs of tumor cells are highly similar between these two model types. Surprisingly, even in the stromal compartment, we detected genes associated with metastatic processes that were upregulated in both PDX models. For instance, ERBB3, a member of the EGFR family known to promote cell survival, proliferation, and metastasis (43, 46–49), showed increased expression in the stromal tissue of ortho PDX models. However, it should be noted that ERBB3’s role in metastasis is not exclusive, as it is also involved in cell proliferation and survival. In contrast, ASPN, which facilitates metastasis by modulating extracellular matrix (ECM) signaling and enabling cancer cell invasion (50), showed significantly increased expression in the stromal tissue of s.c. PDX models.

However, despite the presence of metastasis-associated genes in the stromal compartment of both models, ortho PDX models are generally considered more reflective of true metastatic behavior. One plausible explanation is that, whereas gene expression patterns are similar, the physical and cellular microenvironment of the s.c. space may not fully support the metastatic cascade. The s.c. connective tissues may form a more fibrous, less specialized ECM that impedes invasion and dissemination, effectively creating a physical barrier to metastasis. In contrast, ortho sites provide a native-like ECM composition, vasculature, and signaling milieu that more closely replicate the conditions necessary for metastatic spread, resulting in primary and metastatic tumor growth in ortho PDX models very similar to that of the patient (19, 51).

In light of these observations, for studies focused on characterizing interpatient heterogeneity in cancer transcriptomes, the choice between ortho and s.c. PDX models may not substantially impact research outcomes. Both models capture many aspects of the metastatic gene expression landscape, making s.c. models a cost-effective option when the primary endpoint relates to transcriptional profiling rather than metastatic behavior. The relative ease of monitoring tumor growth kinetics in s.c. models further supports their utility in biomarker discovery (52–54). On the other hand, for studies emphasizing the TME or the functional aspects of metastasis, ortho PDX models should be prioritized due to their closer mimicry of the tumor’s native biological context. This recommendation is bolstered by our identification of numerous DEGs in the mouse (stromal) component, highlighting the importance of tissue-specific microenvironment cues in shaping metastatic potential.

## Supplementary Material

Supplementary Figure 1 Supplementary Figure 1

Supplementary Figure 2 Supplementary Figure 2

Supplementary Table 1 Supplementary Table 1

Supplementary Table 2 Supplementary Table 2

Supplementary Table 3 Supplementary Table 3

Supplementary Table 4 Supplementary Table 4

Supplementary Table 5 Supplementary Table 5

Supplementary Table 6 Supplementary Table 6
