# Supplementary material for: Impact of Subcutaneous Versus Orthotopic Implantations on Patient-Derived Xenograft Transcriptomic Profiles
Source: Cancer Res Commun. 2025 May 28;5(5):871–80. doi: 10.1158/2767-9764.CRC-25-0008 (PMC12117319; doi:10.1158/2767-9764.CRC-25-0008)
Supplement: Supplementary Figure 1 [file crc-25-0008_supplementary_figure_1_suppsf1.docx]

**
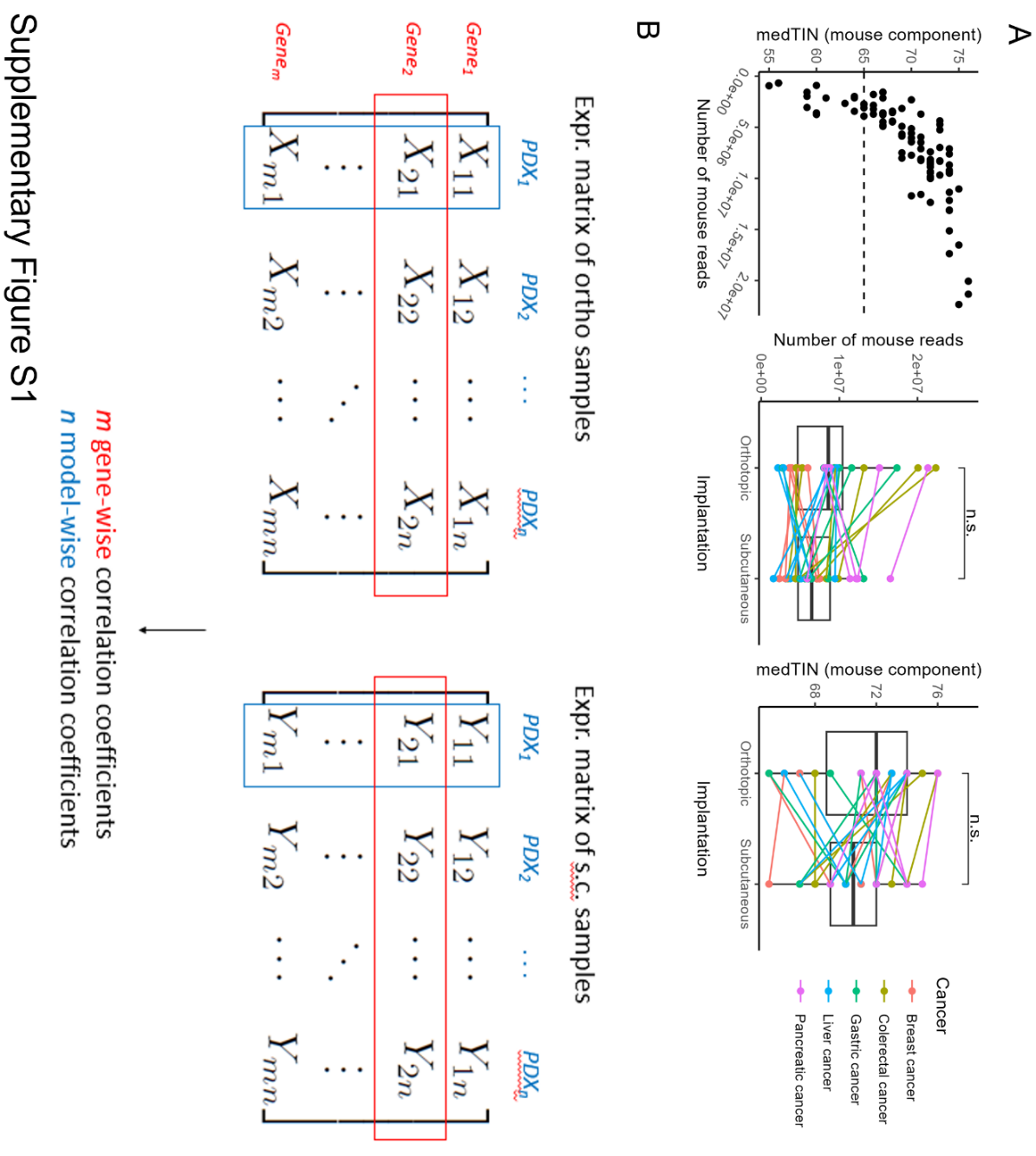
**

**A**. Scatter plot of medTIN scores against mouse read counts in mouse component. Neither the mouse read counts nor the medTIN scores is significantly different between orthotopic and subcutaneous PDX models.

**B**. Diagram of how gene-wise and PDX-wise correlations were performed.
