# Supplementary material for: Impact of Subcutaneous Versus Orthotopic Implantations on Patient-Derived Xenograft Transcriptomic Profiles
Source: Cancer Res Commun. 2025 May 28;5(5):871–80. doi: 10.1158/2767-9764.CRC-25-0008 (PMC12117319; doi:10.1158/2767-9764.CRC-25-0008)
Supplement: Supplementary Figure 2 [file crc-25-0008_supplementary_figure_2_suppsf2.docx]

**
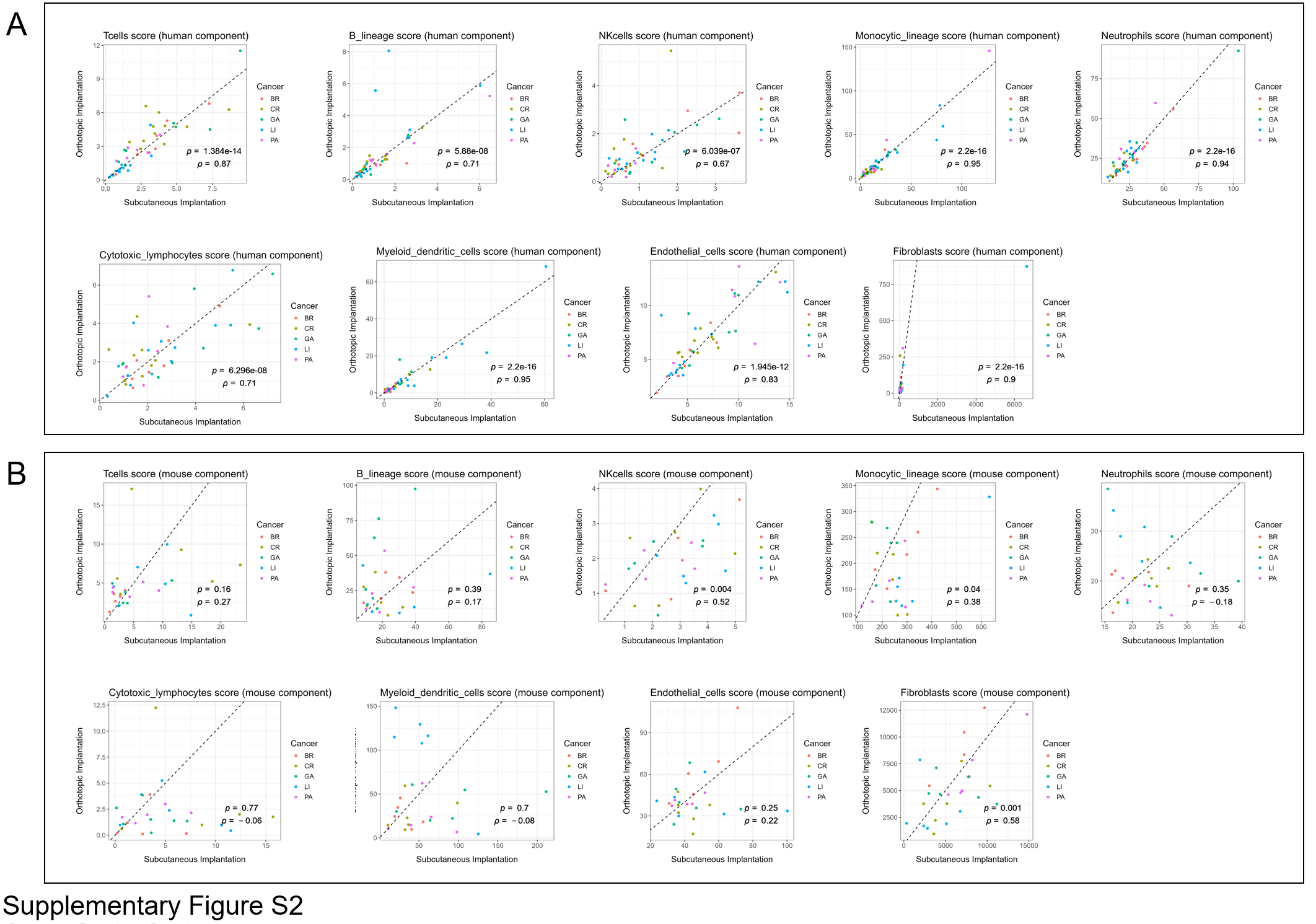
**

**A**. Pearson correlation analysis of MCP-counter different cell scores in the human components between orthotopic and subcutaneous PDX models, with colors representing different cancer types. The dashed line indicates the line of equality (y = x).

**B**. Pearson correlation analysis of MCP-counter different cell scores in the mouse components between orthotopic and subcutaneous PDX models, with colors representing different cancer types. The dashed line indicates the line of equality (y = x).
